# Study of genetic diversity, biofilm formation, and detection of Carbapenemase, MBL, ESBL, and tetracycline resistance genes in multidrug-resistant *Acinetobacter baumannii* isolated from burn wound infections in Iran

**DOI:** 10.1186/s13756-019-0612-5

**Published:** 2019-11-07

**Authors:** Reza Ranjbar, Abbas Farahani

**Affiliations:** 1 Molecular Biology Research Center, Systems Biology and Poisonings Institute, Baqiyatallah University of Medical Sciences, Tehran, Iran; 2 Infectious and Tropical Diseases Research Center, Hormozgan Health Institute, Hormozgan University of Medical Sciences, Bandar Abbas, Iran

**Keywords:** Antimicrobial resistance, OXA carbapenemase, Integron, Biofilm, Clone dissemination

## Abstract

**Background:**

Antimicrobial resistance in multidrug-resistant *Acinetobacter baumannii* (MDR-AB) isolated from burn wound infections is a major concern in intensive care or burns units worldwide, and molecular studies are considered critical strategies for control of MDR-AB outbreaks in this regard. Thus, in this study, antibiotic resistance, biofilm-forming ability, molecular epidemiology of MDR *A. baumannii* strains recovered from patients with burns were investigated in three major hospital centers of Iran.

**Methods:**

In this cross-sectional research, 163 non-repetitive *A. baumannii* strains were tested for susceptibility to antimicrobial agents. Polymerase chain reaction (PCR) was performed to characterize ambler classes A, B, and D β-lactamases, IS*Aba1* and integrons, biofilm formation was also investigated. Clonal relatedness was analyzed using Pulsed-Field Gel Electrophoresis (PFGE).

**Results:**

Among 163 *A. baumannii* strains collected, 94.5% of them were Carbapenem-Non-Susceptible *A. baumannii* (CNSAB) and also 90.1 and 52.2% of them were Metallo-β-Lactamases (MBL) and Extended-Spectrum β-Lactamases (ESBL) producing isolates, respectively. Colistin and polymyxin B exhibited excellent activity against CNSAB strains. High prevalence of *bla*_OXA − 23-like_ (85.1%), *bla*_VIM_ (60.5%), *bla*_PER − 1_ (42.3%), *tetB* (67.8%), and Class 1 integrons (65.6%) were identified in CNSAB strains. IS*Aba1* element was associated with 42 (25.8%) and 129 (98.5%) of *bla*_OXA-51-like_ and *bla*_OXA-23-like_ genes, respectively. 6 clusters with the ability to form strong biofilms were found to be dominant and endemic in our entire areas.

**Conclusions:**

Results of the present study show that antimicrobial resistance in CNSAB isolates from burn wound infections in monitored hospitals in Iran is multifactorial, and also findings of the study suggested that local antibiotic prescription policies should be regularly reviewed, and efficient infection control measures should be observed. Therefore, further strengthening of surveillance of antimicrobial resistance is urgently needed in these regions.

## Background

*Acinetobacter baumannii* is a frequent cause of nosocomial infections in burn patients and has remained an important opportunistic pathogen responsible for increasing rates of morbidity and mortality due to infection in developing countries [1, 2]. Dissemination of MDR *A. baumannii* causes a global public health challenge. Antibiotic treatment of *Acinetobacter baumannii* (*A. baumannii*) infections plays an essential role in reducing prevalence and death rates, but about half of strains of *A. baumannii* in many parts of the world are now resistant to multiple drugs [3]. Prevalence of carbapenemases, cephalosporinase (AmpCs), Extended-Spectrum β-Lactamases (ESBLs), and Metallo-β-Lactamases (MBLs) producing *A. baumannii* is rapidly increasing and conventional antibiotic therapeutics have become increasingly inefficient [3, 4]. Coexistence of various antibiotic resistance mechanisms including extrusion of drugs by active efflux pumps (encoded by various *tet* genes), Carbapenem-Hydrolyzing Class D β-Lactamases (CHDLs) (OXA-23, OXA-24/40, OXA-58, OXA-143, and OXA-235), MBLs (IMP, VIM, NDM, SPM, GIM, and SIM), ESBLs (PER, TEM, SHV,and CTX), contributes to the increase in the number of MDR- AB strains [3, 5]. Multidrug resistance conferred by ESBLs and MBLs genes has recently been raised around the world among Carbapenem-Non-Susceptible *A. baumannii* (CNSAB) strains, and also these resistance genes can be harbored by a transferable plasmid containing integrons or Insertion Sequence (IS) elements into chromosome becoming widely disseminated among other strains, and conferring resistance to almost all β-lactam antibiotics [3, 6]. Recently, much attention has been focused on biofilm formation in *A. baumannii*, because microbial cells grown in biofilms are less sensitive to antimicrobial agents and more persistent to environmental conditions such as intubation tubes, catheters, and cleaning instruments [7, 8].

Drug resistance in CNSAB can result from many mechanisms such as extrusion of drugs by active efflux pumps, decrease in cellular permeability, biofilm formation, and overexpression of drug-modifying and -inactivating enzymes or target modification by mutation [9]. There are few studies conducted in Iran on coexistence of different antibiotic resistance mechanisms with the ability to form biofilms in multidrug-resistant *Acinetobacter baumannii* isolated from burn wound infections, and there is relatively few information on diversity of these strains. Nowadays, understanding molecular characteristics of various antibiotic resistance mechanisms along with molecular epidemiology analysis in regions with high prevalence rates of MDR *A. baumannii* infection plays an essential role in developing therapeutic strategies and controlling MDR-AB outbreaks, both in community and hospitals [1, 7, 10]. Among molecular typing methods, Pulsed-Field Gel Electrophoresis (PFGE) is usually considered gold standard for epidemiological typing of *A. baumannii* [6]. Therefore, this study was carried out to determine antimicrobial susceptibility profiles, biofilm-forming ability, resistance determinants, and clonal relatedness among MDR *A. baumannii* strains recovered from patients with burns in Iran.

## Methods

### Bacterial isolates

In this cross-sectional research, a total of 163 non-repetitive *A. baumannii* strains were recovered from January 2016 to July 2018, from burn wound infections of hospitalized patients in three major hospital centers in Iran, including Markazi (central of Iran, Valiasr Hospital), Khuzestan (southwest of Iran, Taleghani hospital), and Kermanshah (west of Iran, Imam Khomeini hospital).

Isolates were initially identified using the API 20 NE kit (bioMérieux, Marcy-l’Etoile, France) and then confirmed as *A. baumannii* by PCR/sequencing for the intrinsic *bla*_OXA-51-like_ gene described previously [10]. According to the Clinical Laboratory Standards Institute (CLSI), following quality control strains were used as reference strains: *A. baumannii* ATCC 19606, *Pseudomonas aeruginosa* ATCC 27853, and *Escherichia coli* ATCC, 25922.

### Antimicrobial susceptibility testing

Susceptibility to the following antimicrobial agents were determined on the Mueller–Hinton agar (Merck, Germany) by Kirby Bauer disc diffusion method as per Clinical and Laboratory Standards Institute (CLSI, 2017) guidelines [11]: ceftazidime (30 μg), cefotaxime (30 μg), cefepime (30 μg), ceftriaxone (30 μg), tobramycin (10 μg), gentamicin (10 μg), amikacin (30 μg), levofloxacin (5 μg), ciprofloxacin (5 μg), gatifloxacin (5 μg), co-trimoxazole (1.25/23.75 μg), rifampin (5 μg), ampicillin-sulbactam (10 μg/10 μg), piperacillin-tazobactam (100 μg/10 μg), piperacillin (100 μg) (MAST, Group Ltd., Merseyside, UK). The susceptibility testing to imipenem, meropenem, colistin, polymyxin B, doxycycline, minocycline, and tetracycline was carried out using the broth microdilution method according to CLSI instructions and breakpoint criteria [12]. The phenotype(s) of *A. baumannii* isolates were categorized as multidrug-resistant (MDR) when they are “non-susceptibility to at least one agent in three or more antimicrobial categories”, extensively drug-resistant (XDR) means “non-susceptibility to at least one agent in all but two or fewer antimicrobial categories”, and pan drug-resistant (PDR) when they are “non-susceptible to all agents in all antimicrobial categories” [4].

### Phenotypic detection of ESBLs

The isolates were screened for identification of ESBL production by adopting a modified double disc synergy test. As previously described, the antibiotic discs of ceftriaxone (30 μg), ceftazidime (30 μg), cefepime (30 μg), Amoxicillin/clavulanic acid (20/10 μg) and aztreonam (30 μg) were used [13].

### Phenotypic detection of carbapenemase and MBL positive isolates

Carbapenemase production was recognized using modified Hodge test according to Clinical and Laboratory Standard Institute instructions [11]. For the identification of MBL production, all the isolates resistant to imipenem and/or meropenem were tested by E-test MBL strips (AB Biodisk, Solna, Sweden) as per the manufacturer’s instructions.

### Phenotypic detection of efflux pump activity

Efflux pump activity was determined by the broth microdilution method for tetracyclines resistant isolates according to previous studies [14]. MICs of tetracycline, doxycycline, and minocycline were determined in the presence of the following efflux pump inhibitors (EPIs): phenyl-arginine-β-naphthylamide (PAβN) and carbonyl cyanide 3-chlorophenylhydrazone (CCCP) (Sigma-Aldrich Co. St. Louis, USA). A 4-fold or more significant decrease in the MIC values in the presence of EPIs was defined as significant inhibition.

### DNA extraction and polymerase chain reaction assay

DNA templates of isolates were extracted and prepared by QIAamp DNA Mini Kit (Qiagen, Hilden, Germany) according to the manufacturer’s protocol. Concentrations and purity of DNA were determined by NanoDrop one (Thermo Scientific NanoDrop, United States) at 260 nm, and used as a template in PCR technique. Nucleotide sequences of primers used in the study are listed in Table 1.

**Table 1:** Primers sequences used in this study

Primers	Forward sequence	Reverse Sequence	Annealing Temperature (°C)	Expected size (pb)	Reference
OXA-51	TAATGCTTTGATCGGCCTTG	TGGATTGCACTTCATCTTGG	52	353	[10]
OXA-23	GATCGGATTGGAGAACCAGA	ATTTCTGACCGCATTTCCAT	52	501	
OXA-40	GGTTAGTTGGCCCCCTTAAA	AGTTGAGCGAAAAGGGGATT	52	246	
OXA-58	AAGTATTGGGGCTTGTGCTG	CCCCTCTGCGCTCTACATAC	52	599	
OXA-143	TGGCACTTTCAGCAGTTCCT	TAATCTTGAGGGGGCCAACC	52	149	
IS*Aba1*	CACGAATGCAGAAGTTG	CGACGAATACTATGACAC	50	548	
IS*Aba1* + OXA-23	GTGTCATAGTATTCGTCG	ATTTCTGACCGCATTTCCAT	50	875	
IS*Aba1* + OXA-51	CAAGGCCGATCAAAGCATTA	GTGTCATAGTATTCGTCG	50	359	
OXA-253	TTGTTGCCTTTACTTAGTTGC	CAAAATTTTAAGACGGATCG	56	768	[2]
GES	ATGCGCTTCATTCACGCAC	CTATTTGTCCGTGCTCAGG	55	860	[1]
PER-1	ATGAATGTCATTATAAAAG	TTGGGCTTAGGGCAG	45	920	[15]
VEB-1	ATGAAAATCGTAAAAAGGATATT	TTATTTATTCAAATAGTAATTCC	48	900	
CTX − M	ATGATGACTCAGAGCATTCGCCGCT	TCAGAAACCGTGGGTTACGATTTTCG	70	876	
TEM	AAACGCTGGTGAAAGTA	AGCGATCTGTCTAT	45	752	
SHV	ATGCGTTATATTCGCCTGTG	TGCTTTGTTATTCGGGCCAA	60	753	
VIM	TTTGGTCGCATATCGCAACG	CCATTCAGCCAGATCGGCAT	66	500	
GIM	TCAATTAGCTCTTGGGCTGAC	CGGAACGACCATTTGAATGG	53	729	
IMP	GTTTATGTTCATACWTCG	GGTTTAAYAAAACAACCAC	45	188	
SIM	TACAAGGGATTCGGCATCG	TAATGGCCTGTTCCCATGTG	58	741	[16]
NDM − 1	GGTTTGGCGATCTGGTTTTC	CGGAATGGCTCATCACGATC	52	621	[1]
*tet A*	GTAATTCTGAGCACTGTCGC	CTGCCTGGACAACATTGCTT	62	954	
*tet B*	CTCAGTATTCCAAGCCTTTG	ACTCCCCTGAGCTTGAGGGG	57	414	
*intI1*	CAGTGGACATAAGCCTGTTC	CCCGAGGCATAGGCTGTA	55	160	[17]
*intI2*	TTG CGA GTA TCC ATA ACC TG	TTA CCT GCA CTG GAT TAA GC	55	288	
*IntI3*	GCCTCCGGCAGCGACTTTCAG	ACGGATCTGCCAAACCTGACT	62	980	[18]

The PCR reactions were prepared in 20 μl total reaction mixture volume by comprising 10 μl of Taq DNA Polymerase Master Mix Red (Ampliqon, Copenhagen, Denmark), with 3 μl (50 ng) of extracted DNA, and sterile deionized water to achieve a final volume of 20 μl.

The amplifications were carried out in a thermocycler (C1000 Touch, Bio-Rad), with the following conditions: initial denaturation at 95 °C for 1 min, followed by 35 cycles of denaturation at 94 °C, for 30 s, annealing for 40 s (temperature was depending on the nucleotide sequences and mentioned in Table 1), and extension at 72 °C for 90 s with a final extension at 72 °C for 5 min.

Five μl of PCR products were subsequently loaded on a 1.5% agarose gel (Merck Co, Germany) and then stained with SYBR Safe DNA gel stain (Invitrogen), and the DNA bands were visualized and photographed under UV transilluminator (Uvidoc, Gel documentation system, and Cambridge, UK). PCR amplicons were purified by PCR Clean-Up Kit (Vivantis Technologies Sdn. Bhd., Subang Jaya, Malaysia); after that, sent for direct sequencing using an ABI 3730 XL DNA analyzer (Bioneer Corporation, Daejeon, South Korea). All the obtained sequences were trimmed at both the 5′ and 3′ ends and analyzed by the Chromas software version 2.6 and similarity was checked at the NCBI BLAST tool (http://www.ncbi.nlm.nih.gov/BLAST). Also, a positive control for each gene was purchased from the Pasteur Institute of Iran (Karaj, Iran).

### Molecular typing

Genotyping and the clonal relationship between *A. baumannii* strains were determined by pulsed-field gel electrophoresis (PFGE) using *ApaI* restriction enzyme (Thermo Fisher Scientific, USA) for digestion. The Lambda Ladder 48.5–727.5 kb PFG Marker (New England Biolabs, US) was used as DNA size marker. DNA fragments were separated in 1% ultra-pure agarose gels (Invitrogen) using the CHEF Mapper apparatus (Bio-Rad, Munich, Germany) under the following conditions: voltage 6 V/cm; temperature at 14 °C; with switch times ranging from 5 s to 30s; at an angle of 120°, for 19 h. After that, the gels were stained by ethidium bromide, and the DNA bands were visualized and photographed under UV transilluminator. DNA banding pattern images were analyzed using Bionumeric 7.6.3 software (Applied Maths NV, St-Martens-Latem Belgium) and the dendrogram was constructed by UPGMA method (Unweighted Pair Group Method with Arithmetic Mean), based on Dice’s similarity coefficient at a 1.5% tolerance [6]. The similarity of 80% or higher was defined as the same PFGE genotype, whereas the similarity of < 80% indicated various PFGE genotypes.

### Biofilm analysis

Biofilm formation abilities in *A. baumannii* strains were determined by flat-bottomed sterile polystyrene microplates based on the crystal violet staining method according to a previous study [19]. The results were classified into the four following categories: a) OD ≤ ODc = non-biofilm producer; b) ODc < OD ≤ 2ODc = weak biofilm producer; c) 3ODc < OD ≤ 4ODc = medium biofilm producer; d) 4OD < ODc = strong biofilm producer [20].

### Statistical analysis

All statistical analyses performed by SPSS (version 20) (IBM, Chicago, IL, USA). Student’s t-test, chi-square, and Fisher’s exact tests carried out for data analysis. A *P* value of ≤0.05 in all experiments considered statistically significant.

## Results

### Clinical isolates

**Fig. 1:**
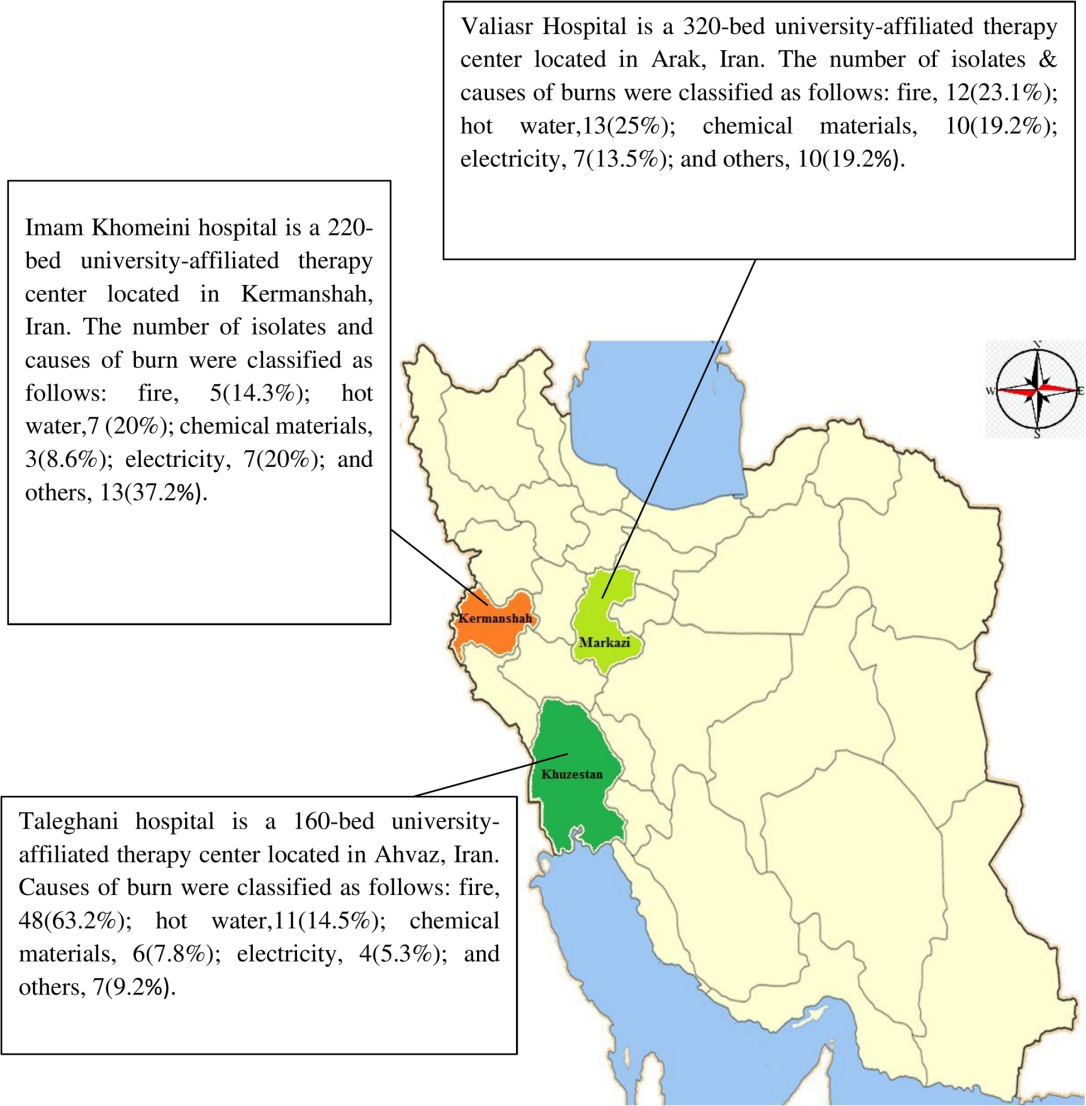
The relative geographical location of each hospital centers are marked in different colors, and the numbers of isolates and constituent ratios are added to the side of the map

Throughout the study period, 163 *A. baumannii* isolates were recovered from burn wound infections in three major hospital centers located in different parts of Iran, including Valiasr Hospital (*n* = 52), Taleghani hospital (*n* = 76), and Imam Khomeini hospital (*n* = 35). The number of different types of burns and relative geographical locations of each hospital centers are represented in Fig. 1. All of the centers have > 160 beds for patients and are the main referral center for burn victims not only for the capital province but also for the neighboring provinces.

### Antimicrobial susceptibility testing

Among 163 clinical isolates of *A. baumannii* recovered from burn wound infections, 89 (54.6%) isolates belonged to male patients and 74 (45.4) belonged to female patients, with a mean age of 29.74 ± 23 years old (Table 2). Out of 163 isolates, 154 (94.5%) isolates were resistant to imipenem and meropenem. Among those isolates, 148/154 (96.1%) revealed positive results on modified Hodge test. Antimicrobial susceptibility testing showed that ≥93% of isolates were non-susceptible to piperacillin, cefepime, ceftriaxone, ceftazidime, cefotaxime, ciprofloxacin, co-Trimoxazole, rifampin, and levofloxacin but high susceptibility to polymyxin B (100%), and colistin (85.9%) (Tables 3 and 4). Among 163 isolates, 140 (85.8%) and 128 (78.5%) of *A. baumannii* strains revealed MDR and XDR phenotypes, respectively. MIC50 and MIC90 for each antibiotic were determined by broth microdilution method (Table 4).

**Table 2:** Distribution of isolates according to demographic characterization

Parameters	Sample proportion (N/%)
Mean age ± SD	29.74 ± 23
Age groups (y)	
0–10	33 (20.2)
11–20	22 (13.5)
21–30	27 (16.6)
31–40	31 (19)
41–50	21 (12.9)
> 50	29 (17.8)
Gender (male/female)	
Male	89 (54.6)
Female	74 (45.4)

**Table 3:** Results of antibiotics resistance profile by disk diffusion among *A. baumannii* isolates

Test Group	Antimicrobial agents	*A. baumannii* antibiogram pattern [*n* = 163 (%)]		
		S	I	R
Penicillins	Piperacillin	6 (3.7)	2 (1.3)	155 (95.1)
β-lactam/ β-lactamase inhibitor combinations	Ampicillin-sulbactam	32 (19.6)	7 (4.3)	124 (76.1)
	Piperacillin-Tazobactam	11 (6.7)	5 (3.1)	147 (90.2)
Cephems	Ceftazidime	3 (1.8)	1 (0.6)	159 (97.5)
	Cefepime	2 (1.3)	8 (4.9)	153 (93.8)
	Cefotaxime	0 (0)	1 (0.6)	162 (99.4)
	Ceftriaxone	0 (0)	0 (0)	163 (100)
Aminoglycosides	Amikacin	13 (7.9)	8 (4.9)	142 (87.2)
	Gentamicin	10 (6.2)	3 (1.8)	150 (92.1)
	Tobramycin	12 (7.4)	2 (1.2)	149 (91.4)
Fluoroquinolones	Ciprofloxacin	4 (2.5)	2 (1.2)	157 (96.3)
	Levofloxacin	8 (4.9)	0 (0)	155 (95.1)
	Gatifloxacin	18 (11.1)	0 (0)	145 (89)
Folate Pathway Inhibitors	Co-Trimoxazole	2 (1.2)	0 (0)	161 (98.8)
Ansamycins	Rifampin	10 (6.2)	0 (0)	153 (93.8)

**Table 4:** Minimum inhibitory concentration (MIC) of seven antimicrobial agents against *A. baumannii* isolates

Antimicrobial agent	MIC (mg/L)			Percent of isolates (%)			Total
	Range	50%	90%	S	I	R	
Imipenem	2–256	32	256	9 (5.5)	0 (0)	154 (94.5)	163
Meropenem	2–256	32	128	9 (5.5)	0 (0)	154 (94.5)	
Colistin	< 0.25–8	2	4	140 (85.9)	0 (0)	23 (14.1)	
Polymyxin B	< 0.25–8	1	2	163 (100)	0 (0)	0 (0)	
Doxycycline	2–256	32	64	17 (10.4)	10 (6.2)	136 (83.4)	
Minocycline	2–256	4	16	57 (34.97)	22 (13.49)	84 (51.53)	
Tetracycline	2–256	32	128	11 (6.7)	6 (3.7)	146 (89.6)	

Abbreviations: *I* intermediate, *R* resistant, *S* susceptible

### Biofilm production

Out of 163 *A. baumannii* isolates, 115 (70.6%) of all *A. baumannii* strains formed a strong biofilm, while 20 (12.2%) and 28 (17.2%) of these isolates were considered as medium and weak biofilm-forming isolates, respectively. Also, our results indicated a significant association of antimicrobial resistance patterns with strong biofilm formation (*P* = 0.001). In addition, a significant association was observed between biofilm-forming ability and XDR phenotype (*P* < 0.05).

### Investigation of resistance mechanisms

*bla*_OXA-51-like_ gene_,_ which is intrinsic to *A. baumannii* was identified in all isolates, and other resistance genes for carbapenems were consisted of 85.1% (131/154) and 54.5% (84/154) of *bla*_OXA-23-like_ and *bla*_OXA-40-like_, respectively. Table 5 represents association between minimum inhibitory concentrations, *bla*_OXA_ carbapenemase genes, and co-existence of them among CNSAB strains. It is worthy to note that; co-occurrence of OXA-23/OXA-40 was present among 70/128 (54.7%) of XDR *A. baumannii* isolates. Other carbapenemase genes such as *bla*_OXA-58-like_, *bla*_OXA-143-like_, and *bla*_OXA-253-like_ were not detected in any of strains. As shown in Table 5, IS*Aba1* element was detected in all CNSAB isolates. Association between IS*Aba1* and three OXA-type carbapenemases revealed that IS*Aba1* element was associated with 42 (25.8%) and 129 (98.5%) of *bla*_OXA-51-like_ and *bla*_OXA-23-like_ genes, respectively, while *bla*_OXA-40-like_ gene had not upstream insertion of IS*Aba1*. A significant increase was observed in MIC values for both imipenem and meropenem among IS*Aba1* + *bla*_OXA-51-like_, and *ISAba1* + *bla*_OXA-23-like_ -carrying isolates. These observations emphasize on *bla*_OXA-23-like_ and *bla*_OXA-51-like_ genes role and their up-regulation by IS*Aba1* element as the major mechanism for carbapenem resistance phenotype.

**Table 5:** The association between the minimum inhibitory concentrations and *bla*_OXA_ carbapenemase genes of 154 isolates that were resistant to imipenem and meropenem

Target genes	Number of isolates with minimal inhibitory concentration of imipenem^a^ and meropenem^b^						*P*-value	No. (%) of isolate
	8 μg/ml	16 μg/ml	32 μg/ml	64 μg/ml	128 μg/ml	256 μg/ml		
*bla*_OXA-23-like_^a^	2 (1.5)	28 (21.4)	41 (31.3)	32 (24.4)	15 (11.5)	13 (9.9)	0.1	131 (85.1)
*bla*_OXA-40-like_^a^	4 (4.8)	9 (10.7)	13 (15.5)	29 (34.5)	19 (22.6)	10 (11.9)	0.2	84 (54.5)
*bla*_OXA-51-like_^a^	10 (6.5)	32 (20.8)	42 (27.3)	35 (22.7)	22 (14.3)	13 (8.5)	0.1	154 (100)
*bla*_OXA-23-like +_ *bla*_OXA-40-like_^a^	2 (2.8)	7 (10)	13 (18.6)	26 (37.2)	13 (18.6)	9 (12.8)	0.1	70 (45.5)
IS*Aba1* + *bla*_OXA-23-like_^a^	0 (0)	28 (21.7)	41 (31.8)	32 (24.8)	15 (11.6)	13 (10.1)	0.1	129 (83.8)
IS*Aba1* + *bla*_OXA-51-like_^a^	0 (0)	2 (4.8)	7 (16.7)	8 (19.1)	12 (28.6)	13 (30.9)	0.2	42 (27.3)
*bla*_OXA-23-like_^b^	1 (0.8)	31 (23.7)	33 (25.2)	29 (22.1)	22 (16.8)	15 (11.4)	0.08	131 (85.1)
*bla*_OXA-40-like_^b^	2 (2.4)	6 (7.2)	15 (17.8)	30 (35.7)	21 (25)	10 (11.9)	0.09	84 (54.5)
*bla*_OXA-51-like_^b^	3 (1.9)	42 (27.3)	34 (22.1)	30 (19.5)	29 (18.8)	16 (10.4)	0.2	154 (100)
*bla*_OXA-23-like +_ *bla*_OXA-40-like_^b^	1 (1.4)	3 (4.3)	11 (15.7)	27 (38.6)	20 (28.6)	8 (11.4)	0.1	70 (45.5)
IS*Aba1* + *bla*_OXA-23-like_^b^	0 (0)	30 (23.2)	33 (25.6)	29 (22.5)	22 (17.1)	15 (11.6)	0.08	129 (83.8)
IS*Aba1* + *bla*_OXA-51-like_^b^	0 (0)	3 (7.2)	5 (11.9)	13 (30.9)	10 (23.8)	11 (26.2)	0.2	42 (27.3)

Abbreviations: ^a^related to the concentration of imipenem; ^b^related to the concentration of meropenem

After screening by ESBL production test, 85 (52.2%) of ESBL-producing isolates were selected for detection of *bla*_VEB_, *bla*_PER-1_, *bla*_GES_, *bla*_CTX − M_, *bla*_TEM_, and *bla*_SHV_. ESBL-producing isolates were non-susceptible to all β-lactams and fluoroquinolones antibiotics. Among all 85 ESBL-producing isolates, *bla*_PER-1_ genes were predominant 55.3% (47/85) followed by *bla*_VEB-1_ 42.3% (36/85)*, bla*_TEM_ 33% (28/85), and *bla*_SHV_ 13% (11/85) (Table 6). *bla*_GES_ and *bla*_CTX − M_ genes were not detected in isolates, and also three (3.5%) ESBLs producing isolates were negative for ESBL encoding genes. Results did not reveal a significant correlation between resistant patterns and presence of ESBL genes in the isolates, but *bla*_PER-1_ and *bla*_VEB-1_ positive strains were predominant among XDR phenotype.

**Table 6:** MBL and ESBL genes detected in *A. baumannii* isolates

Phenotypes and related Genes	Regional profile			Total (%)
	Arak (%)	Kermanshah (%)	Ahvaz (%)	
MBLs	47 (31.9)	32 (21.8)	68 (46.3)	147 (100)
*bla*_VIM_-family	33 (37.1)	17 (19.1)	39 (43.8)	89 (100)
*bla*_IMP_-family	21 (45.7)	7 (15.2)	18 (39.1)	46 (100)
ESBLs	25 (29.4)	19 (22.4)	41 (48.2)	85 (100)
*bla*_PER-1_	14 (29.8)	12 (25.6)	20 (42.6)	47 (100)
푏푙푎_VEB-1_	11 (30.6)	0 (0)	25 (69.4)	36 (100)
*bla*_TEM_	5 (17.8)	9 (32.2)	14 (50)	28 (100)
*bla*_SHV_	0 (0)	8 (72.7)	3 (27.3)	11 (100)
*tetA*	0 (0)	0 (0)	23 (100)	23 (100)
*tetB*	24 (24.2)	27 (27.3)	48 (48.5)	99 (100)

Phenotypic detection of MBL showed that 90.1% (*n* = 147) of isolates were MBL producing isolates. Detection of MBLs by PCR technique revealed that 31.3% (46/147) and 60.5% (89/147) of MBL- producing *A. baumannii* isolates carried *bla*_IMP_- and *bla*_VIM_-family genes, respectively. Also, *bla*_GIM_, *bla*_SIM_, and *bla*_NDM-1_ were not detected in any MBL-producing isolates. In this study, co-existence of *bla*_VIM_ and *bla*_IMP_ genes was observed in 24 strains (16.3%). In addition, tested MBL encoding genes were not detected in 36 (24.5%) of MBLs -producing isolates, and it seems that false-positive results were observed in these strains with respect to resistance phenotype.

Distribution of MBL genes and carbapenemase was similar across all three studied provinces, but ESBL genes such as *bla*_SHV_ and *bla*_VEB-1_ were not found in Arak and Kermanshah provinces, respectively (Table 6).

Class 1 and 2-integrons were identified in 107 (65.6%) and 56 (34.4%) of *A. baumannii* isolates, while class 3 integron was not found. Coexistence of both class 1 and 2-integrons was detected in 39 (24%) of *A. baumannii* isolates. Our results showed a significant correlation between presence of class 1 integron with extensively drug- resistance (*P* = 0.034). In addition, a relationship was found between *bla*_VIM_ gene and class 1 integron in our study (*P* = 0.001).

Susceptibility testing with and without efflux pumps inhibitors showed differences in MIC values and 4- to 8-fold decreases of MICs were observed in presence of efflux pump inhibitors for 87, 90, and 62% of tetracycline-, doxycycline- and minocycline-nonsusceptible *A. baumannii isolates. tetA* and *tetB* genes were recognized in 15.7% (23/146) and 67.8% (99/146) of tetracycline-resistant isolates, respectively (Table 6), however, *tetA* was not detected in any of the isolates recovered from Arak and Kermanshah provinces, and it was not found in any of the minocycline-nonsusceptible strains. Our study showed a significant correlation between presence of *tetB* determinant genes and resistance to minocycline (*P* < 0.05).

### Clonal relationship

**Fig. 2:**
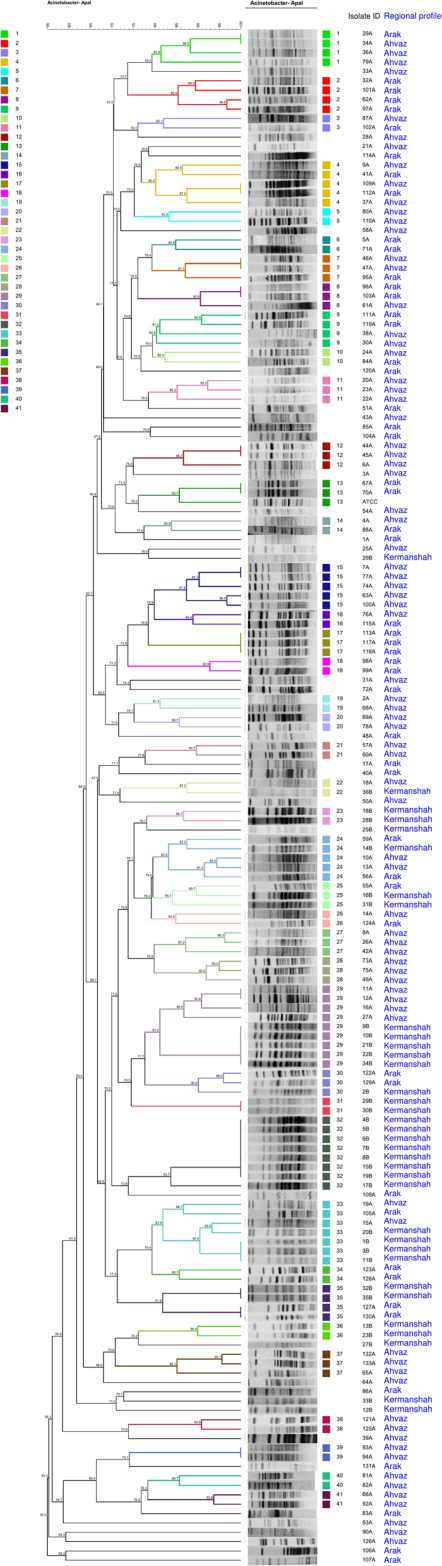
The 41 representative clusters identified by Pulsed-field gel electrophoresis in *Acinetobacter baumannii* isolates using the UPGMA based on Dice similarity

As shown in Fig. 2, PFGE fingerprint revealed polyclonal origins, and all isolates were classified to 41 clusters with 2 or more strains and 33 unique profiles. 6 of these clusters with the ability to form strong biofilms were found to be dominant and endemic in our entire areas, while 24 clusters were sporadic. Clonal distribution of clinical *A. baumannii* isolates was as follows: 14, 6, and 4 clusters were presented in Khuzestan (Ahvaz), Markazi (Arak) and Kermanshah (Kermanshah) provinces, respectively and also 2, 3, and 10 clusters were presented in Khuzestan + Kermanshah, Kermanshah + Markazi, and Khuzestan + Markazi, respectively. Moreover, clusters with numbers of 2, 15, and 32 were dominant only in Markazi, Khuzestan, and Kermanshah provinces, respectively. Also, clinical *A. baumannii* isolates with similar PFGE genotype showed the same pattern of antimicrobial resistance and biofilm formation. In addition, XDR isolates were found to be predominant and showed the same antibiotic resistance genes among cluster numbers of 1, 4, 9, 15, 17, 24, 29, 32, and 33 (Fig. 2).

## Discussion

More than 93% of *A. baumannii* strains obtained from three major hospital centers in Iran were found to be resistant to six antimicrobial categories (Tables 3 and 4). Our study results demonstrated high prevalence rates of CNSAB, MDR-AB, and XDR-AB isolates and emergence of them in respective hospitals as a worrying tendency. In line with other studies conducted in Iran, colistin, and polymyxin B exhibited excellent activity against CNSAB strains [6, 21]. In the present study, high MIC50 and MIC90 values for carbapenems were detected and indicated a markedly reduced efficacy of these agents that could be due to their overuse. In this study, 23 isolates showed resistance to colistin (MIC = 4 ≥ μg/ml), indicating rise of this phenomenon worldwide [22]. This resistance could result from modification of Lipopolysaccharide (LPS) at outer cell envelope, but identification of mechanism of colistin resistance in these isolates was beyond scope of our study [23]. Similar to other studies, our findings showed very high rates of biofilm formation in XDR *A. baumannii* strains [24], and this phenomenon is associated with protective properties of persistent cells in the biofilms,and could be a result of inadequate penetration of antimicrobial agents into the biofilms [24, 25]. Our study results showed a statistically significant association between antimicrobial resistance phenotypes and biofilm formation, which was in line with other findings [9, 24], but it was in contrast to results of the study by Baniya et al. [25]. *A. baumannii* strains formed strong biofilm were endemic in respective hospitals, indicating that microbial cells grown in biofilms are more resistant to different environmental stress conditions [24]. Results of the present study showed that a high distribution of multiple genes, mainly genes of *bla*_OXA-23-like_ / *bla*_OXA-40-like_/ *bla*_OXA-51-like carbapenemase_, *bla*_PER-1_/ *bla*_VEB-1_ extended-spectrum-β- lactamase, *bla*_IMP_- and *bla*_VIM_-family metallo-β-lactamase, and *tetB* efflux pump is responsible for detection of drug-resistance in burn patients. *bla*_OXA-23-like_ and *bla*_OXA-40-like_ are common in CNSAB isolates. Similar to other studies carried out in Iran, *bla*_OXA-23-like_ was the most prevalent OXA-type carbapenemases among carbapenem-resistant strains, [6] and it has reported frequently in many countries including Pakistan, South Korea, and China [26]. According to the data represented in Table 5, increased MIC values observed for imipenem and meropenem in studied *bla*_OXA-23-like_ and *bla*_OXA-40-like_ carrying isolates. The increased MICs indicate that presence of these genes play an important role in creating resistance to carbapenems. Notably, most of the XDR *A. baumannii* isolates co-harbored *bla*_OXA-23-like_ and *bla*_OXA-40-like_ genes, could contribute to clonal dissemination. However, it remains unclear that co-existing carbapenemase genes can cause resistance and it needs further investigation.

However, other carbapenemase genes such as *bla*_OXA-58-like_, *bla*_OXA-143-like_, and *bla*_OXA-253-like_ were not detected in any of the isolates, which was in agreement with previous reports [27]. Results of this study revealed that IS*Aba1 + bla*_OXA-51-like_ and IS*Aba1* + *bla*_OXA-23-like_ -carrying *A. baumannii* isolates had high MIC values for both imipenem and meropenem, and also most resistant patterns were observed to be associated with them (Table 5),and our findings indicate that, this IS element influences expression of antibiotic resistance *bla*_OXA-23-like_ and *bla*_OXA-51-like_ genes, providing higher levels of MIC values to carbapenems,which was in line with results of previous studies [10, 27]. After PFGE analysis, dissemination of clonally related strains was determined in three major hospital centers. Interestingly, all isolates belonging to clusters 24, 29, 32, and 33 were found to be associated with *bla*_OXA_ genes adjacent to IS*Aba1* element, appearing to be predominant clones in monitored hospitals with high similarity in their molecular typing.

Results revealed that 90.1% (*n* = 147) and 85% (52.2%) of isolates were MBL and ESBL producers, respectively, and 43.5% of them were positive for both. It was also found that, ESBL- and MBL- producing *A. baumannii* isolates were genetically related and belonged to the same clone. In the present study, *bla*_PER-1_ genes (55.3%) were predominant followed by *bla*_VEB-1_ (42.3%)*,* and *bla*_TEM_ (33%) among ESBL- *A. baumannii* isolates,which was in accordance with previous studies conducted in Iran and India [28, 29]. On the contrary, Safari et al. reported that 58 and 20% of ESBL- *A. baumannii* isolates harbored SHV, and CTX-M genes, respectively [30]. The *bla*_CTX-M_ gene was not detected in any ESBL producing isolates; higher result was reported (76%) by Abrar et al., 2019 [31]. Also, three (3.5%) ESBLs producing isolates were negative for ESBL encoding genes; however, other mechanisms must be involved in antibiotic resistance of these strains. The *bla*_VIM_- family genes were the most common MBL genotype found in the present study, as reported previously in other studies [32]. On the contrary, *bla*_VIM_- family genes not detected in any MBL-producing isolates from Iran and Egypt [21, 33]. The *bla*_GIM_, *bla*_SIM_, and *bla*_NDM-1_ not identified in the present study; this is in accordance with another report conducted in Iran [30]. There was not any significant correlation between phonotypic MBL and ESBL -producing and resistance genes. In addition, the results obtained by phenotypic identification of MBL production did not comply with genotypic results and indicating that false-positive results among these strains with respect to resistance phenotype. This phenomenon may appear due to effect of the EDTA on cell wall of *A. baumannii* or as chelator to inhibit OXA enzymes [26, 34].

In the current study, 107 (65.6%) and 56 (34.4%) of isolates had class 1 and class 2 integrons, but class 3 integron was not detected. Prevalence of carbapenemases, MBL, and ESBL genes was higher in integron-positive *A. baumannii* strains than in negative strains, and a correlation was found between *bla*_VIM_ gene and class 1 integron (*P* < 0.05) revealing an association of the genes encoding MBL with presence of class 1 integron among MDR-AB isolates. Accordingly, horizontal transfer of this integrons by plasmid promotes spread of multiple resistance genes in sporadic and outbreak isolates of *A. baumannii* [1].

MIC values of tetracycline decreased in presence of efflux pumps inhibitors suggesting involvement of efflux activity. Our findings showed that *tetA* and *tetB* genes were detected in clinical strains with resistance to tetracyclines and doxycycline, which was in agreement with other studies conducted in Spain and Iran [8, 35]. *TetB* was found to be associated with resistance to minocycline, while *tetA* was not found in any of the minocycline-nonsusceptible isolates. These results indicate that *tetB* efflux system might be a potential cause of minocycline resistance, and our results are in concordance with another study conducted by wang et al. [5]. Both of over- expression and lake of *tetB* gene may lead to minocycline-nonsusceptible phenotype [5], but we should consider that other factors and mechanisms could involve in resistance occurrence. All isolates belonging to 29, 32, and 33 PFGE genotypes were found to contain *tetB* gene, possibly leading to clonal dissemination of *tet*(B) positive *A. baumannii* isolates in monitored hospitals. Finally, 15, 17, 24, 29, 32, and 33 PFGE genotypes contained more drug-resistant genes, with the ability to form strong biofilms, and were found to be predominant epidemic clusters, and it is noteworthy that, colistin-resistant *A. baumannii* isolates were widespread in genotypes as mentioned above. Our data obtained from molecular epidemiology analysis is of a significant value for the clinicians to make therapeutic decisions and manage infection control.

## Conclusion

High prevalence of extensively drug-resistant *A. baumannii* isolates in three major hospital centers, with resistance to various classes of antimicrobial agents mediated by efflux pumps activity, and the ability to biofilm formation, as well as presence of integrons, carbapenemases, MBL, and ESBL genes in our study can be a major challenge for treatment with serious implications regarding further spread of resistance genes to other regions. High levels of genetic similarity among MDR-AB species demonstrated wide clonal dissemination in monitored hospitals, which is worrying and requiring efficient and sustained control measures. Overall high-level carriage of antibiotic resistance genes in MDR-AB isolates may have restricted usage of this class of antibacterials as a treatment option and therefore suggesting that local antibiotic prescription policies should be frequently reviewed.

## Data Availability

Not applicable

## Abbreviations

CLSI: Clinical and Laboratory Standards Institute
CNSAB: Carbapenem-non-susceptible *A. baumannii*
ESBL: Extended-spectrum beta-lactamases
MBL: Metallo beta-lactamase
MDR: Multidrug-resistant
PCR: Polymerase chain reaction
PDR: Pandrug resistance
PFGE: Pulsed-field gel electrophoresis
XDR: Extensive drug resistance

## References

[1] Khorsi K, Messai Y, Hamidi M, Ammari H, Bakour R (2015). High prevalence of multidrug-resistance in *Acinetobacter baumannii* and dissemination of carbapenemase-encoding genes blaOXA-23-like, blaOXA-24-like and blaNDM-1 in Algiers hospitals. Asian Pac J Trop Med.

[2] Higgins PG, Pérez-Llarena FJ, Zander E, Fernández A, Bou G, Seifert H (2013). OXA-235, a novel class D β-lactamase involved in resistance to carbapenems in *Acinetobacter baumannii*. Antimicrob Agents Chemother.

[3] Jamal S, Al Atrouni A, Rafei R, Dabboussi F, Hamze M, Osman M (2018). Molecular mechanisms of antimicrobial resistance in *Acinetobacter baumannii*, with a special focus on its epidemiology in Lebanon. J Glob Antimicrob Resist.

[4] Magiorakos AP, Srinivasan A, Carey RB, Carmeli Y, Falagas ME, Giske CG (2012). Multidrug-resistant, extensively drug-resistant and pandrug-resistant bacteria: an international expert proposal for interim standard definitions for acquired resistance. Clin Microbiol Infect.

[5] Wang P, CL ME, Mettus RT, RMQ S, Doi Y. Contribution of the TetB efflux pump to minocycline susceptibility among carbapenem-resistant *Acinetobacter baumannii* strains. Antimicrob Agents Chemother. 2017;61(10):e01176-17. 10.1128/AAC.01176-17 PMC5610478 28739789

[6] Mohajeri P, Farahani A, Feizabadi MM, Norozi B (2015). Clonal evolution multi-drug resistant *Acinetobacter baumannii* by pulsed-field gel electrophoresis. Indian J Med Microbiol.

[7] Eze EC, Chenia HY, El Zowalaty ME (2018). *Acinetobacter baumannii* biofilms: effects of physicochemical factors, virulence, antibiotic resistance determinants, gene regulation, and future antimicrobial treatments. Infect Drug Resist.

[8] Meshkat Z, Salimizand H, Amini Y, Khakshoor M, Mansouri D, Farsiani H (2017). Molecular characterization and genetic relatedness of clinically Acinetobacter baumanii isolates conferring increased resistance to the first and second generations of tetracyclines in Iran. Ann Clin Microbiol Antimicrob.

[9] Singhai M, Rawat V, Goyal R (2013). Concomitant detection of biofilm and metallo-beta-lactamases production in gram-negative bacilli. Indian J Pathol Microbiol.

[10] Kobs VC, Ferreira JA, Bobrowicz TA, Ferreira LE, Deglmann RC, Westphal GA (2016). The role of the genetic elements Bla oxa and IS aba 1 in the Acinetobacter calcoaceticus-*Acinetobacter baumannii* complex in carbapenem resistance in the hospital setting. Rev Soc Bras Med Trop.

[11] Wayne P (2017). Clinical and Laboratory Standards Institute. Performance Standards for Antimicrobial Susceptibility Testing: Twenty seven Informational Supplement M100-S27CLSI.

[12] Testing ECoAS, Testing ECoAS (2017). Breakpoint tables for interpretation of MICs and zone diameters. Version.

[13] Litake GM, Ghole VS, Niphadkar KB, Joshi SG (2015). Phenotypic ESBL detection in *Acinetobacter baumannii*: a real challenge. Am J Infect Dis.

[14] Deng M, Zhu MH, Li JJ, Bi S, Sheng ZK, Hu FS (2014). Molecular epidemiology and mechanisms of tigecycline resistance in clinical isolates of *Acinetobacter baumannii* from a Chinese university hospital. Antimicrob Agents Chemother.

[15] Hujer KM, Hujer AM, Hulten EA, Bajaksouzian S, Adams JM, Donskey CJ (2006). Analysis of antibiotic resistance genes in multidrug-resistant Acinetobacter sp. isolates from military and civilian patients treated at the Walter reed Army medical center. Antimicrob Agents Chemother.

[16] Poirel L, Walsh TR, Cuvillier V, Nordmann P (2011). Multiplex PCR for detection of acquired carbapenemase genes. Diagn Microbiol Infect Dis.

[17] Mirnejad R, Mostofi S, Masjedian F (2013). Antibiotic resistance and carriage class 1 and 2 integrons in clinical isolates of *Acinetobacter baumannii* from Tehran, Iran. Asian Pac J Trop Biomed.

[18] Dillon B, Thomas L, Mohmand G, Zelynski A, Iredell J (2005). Multiplex PCR for screening of integrons in bacterial lysates. J Microbiol Methods.

[19] Wang YC, Huang TW, Yang YS, Kuo SC, Chen CT, Liu CP (2018). Biofilm formation is not associated with worse outcome in *Acinetobacter baumannii* bacteraemic pneumonia. Sci Rep.

[20] Bardbari AM, Arabestani MR, Karami M, Keramat F, Alikhani MY, Bagheri KP (2017). Correlation between ability of biofilm formation with their responsible genes and MDR patterns in clinical and environmental *Acinetobacter baumannii* isolates. Microb Pathog.

[21] Shoja S, Moosavian M, Rostami S, Farahani A, Peymani A, Ahmadi K (2017). Dissemination of carbapenem-resistant *Acinetobacter baumannii* in patients with burn injuries. J Chin Med Assoc.

[22] Hajjar Soudeiha M, Dahdouh E, Daoud Z, Sarkis DK (2018). Phenotypic and genotypic detection of beta-lactamases in Acinetobacter spp. isolates recovered from Lebanese patients over a 1-year period. J Glob Antimicrob Resist.

[23] Trebosc V, Gartenmann S, Totzl M, Lucchini V, Schellhorn B, Pieren M, et al. Dissecting Colistin Resistance Mechanisms in Extensively Drug-Resistant *Acinetobacter baumannii* Clinical Isolates. mBio. 2019;10(4):e01083-19. 10.1128/mBio.01083-19 PMC6635527 31311879

[24] Krzysciak P, Chmielarczyk A, Pobiega M, Romaniszyn D, Wojkowska-Mach J (2017). *Acinetobacter baumannii* isolated from hospital-acquired infection: biofilm production and drug susceptibility. APMIS.

[25] Baniya B, Pant ND, Neupane S, Khatiwada S, Yadav UN, Bhandari N (2017). Biofilm and metallo beta-lactamase production among the strains of *Pseudomonas aeruginosa* and Acinetobacter spp. at a Tertiary Care Hospital in Kathmandu, Nepal. Ann Clin Microbiol Antimicrob.

[26] Uddin F, McHugh TD, Roulston K, Platt G, Khan TA, Sohail M (2018). Detection of carbapenemases, AmpC and ESBL genes in Acinetobacter isolates from ICUs by DNA microarray. J Microbiol Methods.

[27] Meshkat Z, Amini Y, Sadeghian H, Salimizand H (2019). ISAba1/blaOXA-23-like family is the predominant cause of carbapenem resistance in *Acinetobacter baumannii* and Acinetobacter nosocomialis in Iran. Infect Genet Evol.

[28] Vijayakumar S, Mathur P, Kapil A, Das BK, Ray P, Gautam V (2019). Molecular characterization & epidemiology of carbapenem-resistant *Acinetobacter baumannii* collected across India. Indian J Med Res.

[29] Fallah F, Noori M, Hashemi A, Goudarzi H, Karimi A, Erfanimanesh S (2014). Prevalence of Bla NDM, Bla PER, Bla VEB, Bla IMP, and Bla VIM genes among *Acinetobacter baumannii* isolated from two hospitals of Tehran. Iran Scientifica.

[30] Safari M, Mozaffari Nejad AS, Bahador A, Jafari R, Alikhani MY (2015). Prevalence of ESBL and MBL encoding genes in *Acinetobacter baumannii* strains isolated from patients of intensive care units (ICU). Saudi J Biol Sci.

[31] Abrar S, Ain NU, Liaqat H, Hussain S, Rasheed F, Riaz S (2019). Distribution of bla CTX - M , bla TEM , bla SHV and bla OXA genes in Extended-spectrum-beta-lactamase-producing Clinical isolates: A three-year multi-center study from Lahore, Pakistan. Antimicrob Resist Infect Control.

[32] Amudhan MS, Sekar U, Kamalanathan A, Balaraman S (2012). blaIMP and blaVIM mediated carbapenem resistance in Pseudomonas and Acinetobacter species in India. J Infect Dev Ctries.

[33] Alkasaby NM, El Sayed Zaki M (2017). Molecular Study of *Acinetobacter baumannii* Isolates for Metallo-beta-Lactamases and Extended-Spectrum-beta-Lactamases Genes in Intensive Care Unit, Mansoura University Hospital, Egypt. Int J Microbiol.

[34] Bedenic B, Ladavac R, Vranic-Ladavac M, Barisic N, Karcic N, Sreter KB (2019). False positive phenotypic detection of metallo-beta-lactamases in *Acinetobacter baumannii*. Acta clinica Croatica.

[35] Roca I, Espinal P, Marti S, Vila J (2011). First identification and characterization of an AdeABC-like efflux pump in Acinetobacter genomospecies 13TU. Antimicrob Agents Chemother.

